# AI health literacy as a foundation for responsible AI in healthcare: a framework for trustworthy implementation

**DOI:** 10.3389/fdgth.2026.1832356

**Published:** 2026-07-21

**Authors:** Olga Tzortzatou Nanopoulou

**Affiliations:** Digital Health Literacy and Policy Hub, Nanopoulos Foundation, NY, United States

**Keywords:** AI health literacy, algorithmic bias, artificial intelligence ethics, clinical decision support, data governance, digital health literacy, eHealth literacy, health equity

## Abstract

**Background:**

Artificial intelligence (AI) is rapidly transforming how healthcare is delivered, but there is mounting evidence revealing a critical gap between technical sophistication and the human capacity to deploy, interpret, and govern these systems responsibly. Current ethical AI frameworks emphasize fairness, transparency, and accountability but they miss a foundational prerequisite: stakeholder AI literacy.

**Objective:**

This paper proposes a conceptual framework positioning digital health literacy, specifically AI-augmented health literacy, as the measurable literacy competencies among clinicians, patients, and governance professionals, even in ethically designed AI systems will fail to achieve trustworthy deployment.

**Methods:**

This paper is a critical narrative review with a normative governance purpose. This paper integrates evidence from three domains (1) documented AI failures in healthcare due to literacy gaps (2) systematic reviews of digital health literacy measurement tools revealing their inadequacy in the context of AI (3) insights from European data governance (General Data Protection Regulation [GDPR], European Health Data Space [EHDS] and AI Act) illustrating that legal harmonization without stakeholder comprehension produces fragmented, ineffective governance.

**Results:**

The analysis of high-profile AI implementation failures suggest a recurring pattern in which literacy deficits at critical decision points may have been a contributing factor. Current measurement tools, including the eHealth Literacy Scale (eHEALS, developed 2006), lack AI-specific competencies and measure perceived instead of actual skills. We identify six AI literacy dimensions lacking in existing instruments, namely algorithmic literacy, bias awareness, data governance understanding, critical appraisal of AI outputs, trust calibration, and explainability interpretation.

**Conclusions:**

These findings suggests that responsible AI in healthcare may require concurrent investment in human capacity building alongside technical and regulatory development. This paper proposes a three-tier literacy framework, encompassing clinician AI literacy, patient AI literacy, and governance AI literacy, with sector-specific competencies and assessment strategies. The three-tier framework proposed here is offered as a governance hypothesis requiring empirical validation rather than as a conclusion already established by the available evidence.

## 1 Introduction

### 1.1 The Gap between governance and understanding

The speed at which healthcare systems have adopted artificial intelligence has outpaced our ability to govern it effectively. AI-powered clinical decision support tools can now analyze medical images, anticipate sepsis onset, triage emergency presentations, and generate treatment recommendations. However, while organizations repeatedly deploy technically sophisticated and regulatory-compliant systems into clinical environments, the human beings responsible for using, overseeing, and governing those systems lack the foundational competencies to do so safely (1, 2).

Three well-documented implementation failures exemplify this pattern and are analyzed in Section 3. First, a sepsis prediction algorithm widely deployed in hospital settings was found to perform poorly at standard alert thresholds, raising questions regarding the pre-deployment evaluation phase (3). Second, a care management algorithm was shown to systematically underestimate the health needs of Black patients, which was due to structural inequities built into its cost-based predictions (4). Finally, an oncology AI system was reported to have generated unsafe or incorrect treatment recommendations in internal assessments before these concerns became publicly known (5). What these failures may share in common is not simply a technical deficit but a plausible governance one as well, where there is compliance without understanding. Healthcare organizations conducted numerous risk impact assessments, secured ethics approvals, and documented rigorously deployment protocols, yet the individuals responsible for operating these systems did not adequately understand the system they were using or how they should evaluate whether it was working or not.

### 1.2 The European data governance lesson

Europe's experience with data protection legislation offers an instructive lesson. In theory, the General Data Protection Regulation (GDPR) was meant to harmonize data protection standards across all European Union Member States. However, when it came to health data processing, it produced precisely the opposite: the fragmentation it was meant to prevent. In the field of biomedical research for instance, biobanks, large collections of health data and samples, which were operating under identical legal provisions across the EU, began to adopt divergent consent practices. Moreover, ethics committees conflated data access with data transfer when evaluating federated learning proposals, while data protection officers (DPO's) demanded additional consent because they misunderstood the legal bases for processing under the GDPR, Article 6(1) read in conjunction with Article 9(2)(j) and the research safeguards of Article 89(1) as a requirement for explicit individual authorization (6).

Furthermore, the European Health Data Space [EHDS; Regulation (EU) 2025/327, published in the Official Journal of the European Union on March 5, 2025 and entering into force on March 26, 2025] represents a further attempt to achieve the long awaited harmonization through the standardized data permits, Health Data Access Bodies and technical interoperability infrastructure. The Regulation does not become generally applicable on entry into force. The European Commission is required to adopt key implementing acts by March 26, 2027. Primary use provisions including the cross-border exchange of patient summaries and ePrescription as well as secondary use rules for most electronic health record data categories, will begin to apply on March 26, 2029 and the second group of primary data categories, encompassing medical imaging, laboratory results, and hospital discharge reports, will become applicable across all Member States on March 26, 2031. Yet the GDPR experience demonstrated that legal uniformity does not guarantee interpretive consistency. The same fragmentation that undermined on a European level GDPR's promise, now threatens to repeat itself in global AI governance unless the literacy foundation, the shared human capacity to interpret and apply regulatory frameworks with critical judgment, is systematically built alongside the technical architecture of EHDS (7).

### 1.3 Digital health literacy in the context of AI

Digital health literacy, which is defined as “*the ability to seek, find, understand, and appraise health information from electronic sources and apply the knowledge gained to addressing or solving a health problem*”, has been mostly measured through the eHEALS (8). Developed in 2006, this eight-item tool predates the smartphone, the patient portal, wearable health devices, chatbots and all modern AI applications in clinical care. Validation studies have shown that the eHEALS assesses perceived competence rather than demonstrated performance, with respondents consistently overestimating their own skills (9).

A 2025 mixed-methods study evaluating existing digital health literacy tools concluded that eHEALS is limited in scope and adaptability for modern contexts, suggesting that future measuring instruments should be designed to capture emerging digital competencies, including individual skills, context-specific factors, health system considerations, specific technologies employed, and user interface dimensions (10). These constraints point toward the need for a conceptually distinct term, AI health literacy, defined here as the skills required to critically understand, assess and safely act upon outputs generated by AI systems in healthcare contexts. AI health literacy, unlike health literacy or digital health literacy, includes algorithmic reasoning, bias recognition, trust calibration, and data governance comprehension all skills which are absent from the current validated instruments (11, 12). No validated instrument currently assesses AI health literacy competencies. This indicates a significant measurement deficiency, since AI systems are being deployed at scale without any validated methods to ascertain whether the clinicians, patients, and governance professionals involved possess the necessary skills to use them safely. Table 1 summarizes the conceptual distinctions between the three terms as used in this paper.

**Table 1:** Conceptual distinctions Between Health Literacy, Digital Health Literacy, and AI Health Literacy.

Dimension	Health literacy	Digital health literacy	AI health literacy
Core definition	Ability to obtain, understand, and use health information to make informed decisions	Ability to seek, find, evaluate, and apply health information from electronic sources	Ability to critically understand, evaluate, and safely act upon AI-generated outputs in healthcare
Primary domain	Clinical information and navigation	Online health information and digital services	Algorithmic systems, LLMs, clinical decision support, AI-generated documentation
Key competencies	Reading comprehension; provider communication; self-management	Online search; portal navigation; privacy awareness	Algorithmic literacy; bias recognition; trust calibration; explainability interpretation; data governance understanding
Typical measurement tool	Newest Vital Sign; REALM; TOFHLA	eHEALS; DHLI	No validated instrument currently exists
Year of leading instrument	1993–2005	2006–2017	—
Covers AI-specific risks	No	No	Yes
Covers hallucination recognition	No	No	Yes
Covers algorithmic bias awareness	No	No	Yes
Covers governance competencies	No	Partially	Yes

### 1.4 Research goals

This paper proposes that AI failures in healthcare may in significant part be attributed to literacy deficiencies at critical decision-making junctures, and that this explanatory pathway remains under-investigated. The claim is advanced as a testable governance hypothesis rather than an established empirical finding. Current digital health literacy frameworks are inadequate for AI and need to be substantially expanded to include skills in algorithmic literacy, bias awareness, and data governance competencies. Most importantly, literacy needs to be thought of not as another “training add-on program” but as an infrastructural requirement.

### 1.5 Methodology

This paper uses a theory-driven narrative review approach to synthesize information across three domains and to develop a conceptual governance framework. This strategy is appropriate when the evidence base for a topic is insufficient for comprehensive meta-analysis, and where the primary academic contribution lies in framework construction rather than quantitative synthesis (13). Thus, the paper is best characterized as a critical narrative review with a normative governance purpose since it does not aim to provide a comprehensive balanced survey of all available literature. Its main objective is to build and justify a three-tier AI health literacy framework by synthesizing empirical evidence, regulatory analysis, and measurement scholarship.

The research was conducted using PubMed, Google Scholar, Scopus, and SSRN, and supplemented with searches on legislative databases (EUR-Lex, HHS regulatory archives) using the following term clusters: (artificial intelligence OR machine learning OR large language model) AND (health literacy OR digital health literacy OR AI literacy), AI implementation failure OR algorithmic bias healthcare, eHEALS OR DHLI OR health literacy measurement, GDPR health data fragmentation, EHDS governance, EU AI Act Article 4 AI literacy, clinical decision support oversight. The searches were carried out from the beginning of the research up until April 2025, with an emphasis on publications from 2018 to 2025 given the recency of the topic. Foundational earlier sources (e.g., eHEALS 2006) are also included when they constitute the authoritative primary reference.

Peer-reviewed empirical studies, systematic and narrative reviews, and commentary articles on AI implementation, health literacy, digital health literacy measurement or AI governance in healthcare contexts, as well as European legislative and regulatory texts (GDPR, EHDS Regulation and EU AI Act) are cited as primary legal sources according to doctrinal methods standard in health law research. Case studies on AI implementation with documented peer-reviewed or credible journalistic records were also included. Legal and policy sources are regarded descriptively as documentary evidence rather than as empirical data.

The three AI implementation cases discussed in Section 3.1 were selected because they are among the most thoroughly documented in the peer-reviewed or credible investigative literature, span different institutional contexts (hospital, population health management, oncology), and involve different failure mechanisms, permitting a comparative literacy-focused reading. They are not to represent a typical sample or prove a causal relationship but are offered as illustrative examples.

A variety of evidence types and weights were applied in the analysis. The strongest claims are backed by peer-reviewed empirical studies and systematic reviews (Sections 3.2, 4.1). The case study studies in Section 3.1 are based on a combination of peer-reviewed publications (3, 4) and credible investigative journalism (5). The IBM Watson case is included as it is the most thoroughly documented public example of a high-profile governance failure in oncology AI. However, IBM disputed elements of the reporting and the case should be read as illustrative of a governance pattern and not an established causal account. Claims based on journalistic sources are carefully qualified throughout. The examination of current digital health literacy tools (Section 4) focuses on the most cited and validated instruments (eHEALS, DHLI, AIPeHLS, modified eHEALS) as recognized in the latest systematic review of the topic (10). It should be noted that a thorough systematic audit of all current instruments has not been carried out and certain instruments may partially address some of the 6 specified dimensions.

Artificial intelligence support (Claude/Anthropic) assisted with literature search verification, reference formatting and manuscript editing. All analytical content, interpretive frameworks, arguments and conclusions are the author's own.

### 1.6 Evidence hierarchy note

The analysis utilizes evidence of diverse forms and strengths. The most robust claims are supported by peer-reviewed empirical research and systematic reviews (Sections 3.2 and 4.1). The case study analyses in Section 3.1 draw on a combination of peer-reviewed publications (3, 4) and credible investigative journalism (5). The IBM Watson case is included because it represents the most comprehensively documented public account of a significant governance failure in oncology AI. However, IBM disputed elements of the reporting and the case should be interpreted as indicative of a governance pattern rather than as an established causal account. Assertions originating from journalistic sources are explicitly hedged throughout. Legal analysis relies on main legislative sources and is regarded as doctrinal rather than empirical evidence.

## 2 Theoretical framework: literacy as infrastructure

### 2.1 The AI literacy stack

This paper proposes a heuristic conceptual model for AI health literacy based on a hierarchical competency stack. This model is not empirically validated and should not be used to imply that the developmental sequence depicted is empirically necessary. Whether higher-layer competencies such as AI augmented literacy and governance literacy genuinely require lower-layer foundations (health literacy, digital health literacy) as a prerequisite remains an open empirical question. The hierarchy is offered as a theoretically motivated organizing framework to structure competency development priorities and guide instrument design analogous to the way that Bloom's taxonomy was used as a heuristic before its empirical structure was investigated. AI health literacy is proposed as a context-specific construct that is conceptually related to, but qualitatively distinct from health literacy and digital health literacy as currently operationalized. It requires competencies such as adversarial hallucination recognition, fairness metric interpretation, and dataset shift detection that have no operational parallel in already validated instruments.

The base layer refers to basic health literacy which means acquiring information about health conditions and treatment options, navigating healthcare facilities, communicating effectively with healthcare providers, and supporting personal health management (14). The second layer, digital health literacy, goes further by including the ability to find and critically evaluate online health information, use patient portals and telehealth platforms, understand the implications of data privacy and consent, and interact with digital health services with confidence (8). Sørensen et al. have argued that large-scale improvement in health literacy at this level requires not only individual skill development but systemic investment in organizational structures, resources, and political commitment across healthcare settings (15).

The third layer, AI-augmented health literacy, requires competencies that go far beyond general digital skills. For example, to understand the difference between algorithmic predictions and clinical diagnoses, be able to identify potential sources of algorithmic bias, be able to tell the difference between AI-generated and human-generated content, and finally, be able to calibrate trust appropriately. These competencies are synthesized from responsible AI frameworks, including those proposed by Floridi and colleagues (16) and the EU High-Level Expert Group on AI (17), in addition to existing empirical work done on automation bias and trust calibration in clinical decision support (1, 2). The fourth layer, data governance literacy, is primarily for professionals. It includes knowing how to use privacy-preserving techniques such as federated learning and understanding privacy concepts such as the distinction between de-identification and anonymization of personal data, being aware of re-identification risks, auditing fairness metrics, and translating technical safeguards and technical measure, into policy-relevant language. These competencies correspond to stipulations delineated in data governance research and European health data policy (6).

This proposed hierarchy presents a plausible, albeit unvalidated, explanation for the frequent failures in current AI implementation. Deployment may be striving to cultivate third- and fourth-layer competencies among stakeholders whose second-layer digital health literacy foundation is itself inadequately developed or assessed (9, 10). The question of whether the hierarchical structure of this model accurately represents the development of literacy competencies in practice, is one of empirical nature, requiring empirical investigation.

### 2.2 Three critical areas of AI health literacy

#### 2.2.1 AI literacy for clinicians

Essential clinician competencies include the interpretation of model validation metrics (sensitivity, specificity, positive predictive value, negative predictive value, area under the receiver-operating-characteristic curve), comprehension of confidence intervals and calibration, identification of instances where AI hallucinates and extrapolates beyond its training data, auditing alerts for demographic performance disparities, critical evaluation of vendor claims against peer-reviewed validation, and understanding of fatigue alert.

The current literature on AI adoption among healthcare professionals is primarily characterized by studies on acceptance and willingness to use AI tools, rather than on comprehension or ability to critically evaluate model performance (1, 2). Skills that would be essential for independent evaluation such as the ability to interpret external validation statistics or check for differences in performance between different groups of people are seldom required and tested. A closely related challenge to the above is dataset shift: when an AI model is deployed in a patient group that significantly differs from the group it was trained on, its ability to make accurate predictions can drop substantially. Finlayson et al. have identified this as a fundamental and underappreciated challenge for clinicians utilizing AI tools, who may lack the literacy required to discern when a model is employed outside the validated conditions of its performance (18).

#### 2.2.2 AI literacy for patients

Van Kessel et al. have suggested that digital health literacy may serve as a super-determinant of health, a factor that influences access to and use of a wide array of health-related resources and services across multiple domains, while explicitly recognizing that the empirical validity of this relationship is yet to be determined (19). However, many commonly used patient digital portals now display AI-generated summaries, risk scores, and diagnostic flags as a normal part of care. Patients, who rarely get support in making sense of these outputs, when they interact with AI mediated care, need skills which go well beyond health literacy or digital health literacy. Those skills include among others the capacity of interpreting AI-flagged results, understanding risk scores and probabilistic predictions, recognizing when AI outputs need human confirmation, being able to recognize AI limitations in personalized contexts and understanding how their data may be reused in AI training. A review of patient perspectives on AI in medical imaging revealed that numerous patients lack awareness of the integration of AI into their clinical care, raising significant concerns about their capacity to interpret AI-generated outputs or differentiate them from clinician judgments (20). Using AI without also investing into patient literacy programs could worsen the existing inequities among people of different ages, education, language, and socioeconomic status.

#### 2.2.3 AI literacy for governance

Core governance competencies include comprehending privacy-preserving techniques and their limitations, distinguishing between data access and data transfer in federated systems, identifying contemporary privacy attacks (membership inference, model inversion), interpreting fairness metrics (demographic parity, equalized odds, calibration), translating between legal compliance and technical safeguards, and evaluating vendor claims about explainability and transparency.

The US Department of Health and Human Services Secretary's Advisory Committee on Human Research Protections (SACHRP) has officially recognized a structural governance deficiency in the oversight of AI research: a significant portion of AI and machine learning research falls outside of the Common Rule's definition of “human subjects research”, meaning that it may comply with the existing regulatory standards while failing to adequately safeguard the participating individuals whose data are being processed (21). SACHRP recommended that the HHS Secretary revisits the regulatory definition of identifiability and make formal guidance to address the new risks that AI research introduces, such as group harms and re-identification risks.

Because of this compliance protection gap, an ethics review of AI projects might be able to meet its regulatory obligations without examining model validity, demographic fairness, or generalizability. Governance professionals often mix up different legal concepts, such as in the case of GDPR interpretation analyzed above where the legal basis for processing under GDPR, was mixed with the with consent requirements. This eventually causes procedural delays that waste institutional resources without really enhancing participants' rights and offering any additional protection to the latter (6).

## 3 When literacy gaps meet algorithms

### 3.1 Case study analysis

The cases below have been selected because they are three of the most well-documented failures of AI deployment in the peer-reviewed or reliable investigative literature, and because each has a different institutional setting and mechanism of failure. They are not meant to show literacy deficits as established causal factors of failure as the available evidence does not support such a conclusion. The aim of this analysis is to investigate whether a literacy-based analytical lens can reveal governance vulnerabilities that existing systemic explanations have not fully examined. The following literacy interpretations are the author's own analysis and not the result of statements endorsed by the sources listed. They are offered as governance hypotheses to be explored empirically, not conclusions derived from the facts discussed in this article. This framework is relevant for all case study analyses in this paper.

#### 3.1.1 Sepsis prediction algorithm

An independent external validation study of a widely used proprietary sepsis prediction algorithm found that the AI tool did not perform well at its standard alert levels. That caused a substantial number of false alarms while eventually failing to accomplish its original goal, which was to help the hospital staff identify a significant number of sepsis cases (3). Wong et al. reported in their study that the algorithm continued to be utilized routinely across numerous institutions despite its performance profile and raised concerns about the sufficiency of the evaluation processes that had preceded the tool's deployment in the hospital settings.

From a literacy perspective, the failure can be interpreted through three interrelated levels. At the deployment level, hospital administrators may not have had the technical skills to independently evaluate vendor claims about the tool's performance before deciding to roll out the system across the board. At the clinical level, doctors and nurses interpreting the tool may not have known how to balance sensitivity and specificity, nor how the local rate of sepsis would change the alerts they were receiving. At the governance level, safety committees seem to have approved the tool's deployment without requiring either independent external validation or performance monitoring broken down by patient demographic characteristics. The exact role of these potential literacy gaps, whether they were causal or just contextual, cannot be established from the available evidence. However, they are presented here as possible reasons that warrant further investigation.

#### 3.1.2 Bias in care management

Obermeyer et al. reported that a prevalent care management algorithm utilized healthcare costs as a surrogate for health needs in the identification of high-risk patients for targeted interventions. This revealed a methodology that systematically underestimated the needs of Black patients at the study institution (4). More specifically, due to historical disparities in healthcare access for Black patients, stemming from entrenched systemic inequities, their reduced healthcare costs were inaccurately interpreted by the algorithm as indicative of lower clinical need. The authors explicitly stated that their findings are based on a single algorithm used at one academic medical center and may not apply to other implementations.

Read through a literacy lens, the failure suggests gaps operating at three levels: data scientists may not have realized how deeply structural inequities in healthcare access would spread through a cost-based proxy, ethics and compliance teams may not have had the grounding in fairness metrics necessary to audit the algorithm for demographic disparities and clinicians using the tool may not have known that a Black patient's apparently low predicted risk score could be an artifact of systemic undertreatment rather than a reflection of their actual health status. Notably, Obermeyer et al. developed at a later stage a corrected algorithm that made risk prediction much fairer and more accurate (4).

#### 3.1.3 AI-assisted decision support for cancer treatment

A STAT News investigation drawing on internal IBM documents reported that an oncology AI system had made treatment recommendations which internal reviews said were unsafe or incorrect in certain cases. The system had already been used across hospitals in several countries before those concerns became public (5). IBM disputed elements of the reports against them, and the system was eventually absorbed when IBM Watson Health was sold to a private equity firm in 2022. Read through a literacy lens, the case shows what might be called a “market literacy gap”: oncologists and hospital procurement teams may have lacked the AI-specific literacy needed to distinguish marketing claims (such as whether the specific tool was trained solely on medical literature) from the kind of evidence of prospective clinical validation that would normally be required before a pharmaceutical agent could be adopted by a healthcare provider. It is not possible to determine from the available source whether literacy deficits influenced adoption decisions at particular institutions since the question of AI literacy in clinical procurement remains largely uninvestigated in the empirical literature.

### 3.2 Typical failure patterns

The three cases above differ from one another in their causes, institutional contexts, and severity. The literacy interpretations given throughout are the author's analytical framing. However, even with that qualification in mind, cross-case analysis raises five hypothetical patterns that may be worth investigating in future literacy-focused research. First, trust seems to have been misplaced. In each case stakeholders relied on vendor assurances, the institution's reputation, or the fact that the system had been approved by regulators instead of critically appraising and using their own judgment ability for the systems they were using. Second, it seems that governance was more about “following the rules” and, while the required review processes were finished, there was no real discussion of the technical issues of model validity, generalizability, or demographic performance. Third, there was a literacy asymmetry in each case: the AI systems were much more advanced than the people who oversaw using and monitoring them. Fourth, none of the three cases reported a pre-deployment evaluation of stakeholder literacy skills. Fifth, literacy gaps seem to have spread and problems with governance seem to have led to conditions that brought about clinical deployment failures. This paper cannot provide answers as to whether these patterns are indicative of a broader category of AI implementation failures, but the analysis suggests that this is a significant area for future research.

#### 3.2.1 Generative AI and new ways things might go wrong

The three case studies presented in the above Section 3.1, pertain to predictive and rule-based algorithmic systems and predate the extensive use of generative AI in clinical settings. Nonetheless, the same five failure patterns identified across those cases misplaced trust in vendor assurances, procedural compliance without substantive understanding, asymmetry between system sophistication and user competency, absence of pre-deployment literacy assessment, and cascading governance deficits are documented in the growing literature on large language models (LLMs) in the field of healthcare. In fact, in many ways, the risks may be even greater: LLM outputs are linguistically fluent, contextually plausible, and often indistinguishable from content which is created by experts.

#### 3.2.2 Hallucinations in clinical documentation

A critical issue in the deployment of LLMs for clinical documentation and recordkeeping is the potential that outputs may contain hallucinations; that is, clinically relevant inaccuracies with no basis in the original data. Asgari et al. conducted what they describe to be the largest manual evaluation of LLM clinical note generation to date, examining 12,999 clinician-annotated sentences across 18 experimental configurations. They identified a hallucination rate of 1.47% and an omission rate of 3.45%, with 44% of hallucinations deemed significant, meaning they could affect patient diagnosis or management if not corrected (22). The most prevalent hallucination types were fabrication (43%), negation (30%), and contextual misinterpretation (17%). These forms of hallucinations occurred most frequently in the plan, evaluation, and symptoms sections of notes, which are precisely the sections most likely to influence subsequent clinical decision-making. The authors noted that conventional automated evaluation measures like ROUGE and BLEU are insufficient for detecting semantically significant clinical errors, a finding that raises concerns about whether institutions using ambient AI scribes possess the tools to independently evaluate the safety of the outputs they are approving.

This issue is explicitly discussed in a commentary by Topaz et al., which highlights various dangers linked to the swift integration of AI scribes within the healthcare systems, noting that the speed of adoption has surpassed validation, transparency, and regulatory oversight, with concerns raised about the quality and trustworthiness of AI-generated clinical information (23). They also stress that private opacity of vendor AI engines complicates independent safety evaluations, reflecting the same governance literacy gaps which were identified in the IBM Watson case (Section 3.1.3). The authors also expressed a particular concern that the speech recognition systems used by AI scribes are less accurate when transcribing the speech of Black patients in comparison to white patients. This discrepancy may lead to systematically deficient documentation for patients from marginalized communities, exacerbating health inequities similar to those identified by Obermeyer et al. (22, 23).

#### 3.2.3 Adversarial hallucination and the limits of training

A significant difficulty for AI health literacy is the vulnerability of LLMs to adversarial hallucination: the propensity to expand upon, rather than rectify, false facts present in clinical vignettes. Omar et al. developed 300 physician-validated clinical vignettes, each incorporating one false detail, a laboratory value, a physical sign, or a medical condition. They then evaluated six LLMs across 5,400 outputs (9). Hallucination rates varied between 50% and 82% across different models and prompting techniques, with a baseline average of 66%. Prompt-based mitigation techniques decreased the mean rate to 44%, but did not eradicate the risk, while temperature modifications provided no notable enhancement. The authors assert that LLMs are particularly vulnerable to adversarial hallucination when utilized in clinical settings for decision support, lacking sufficient safeguards, and that their conclusions bear considerable implications for patient safety. This finding is directly pertinent to the fundamental inquiry regarding the adequacy of AI literacy training as a protective measure. Evidence of adversarial hallucination indicates that even when doctors are educated to recognize LLM errors, the linguistic plausibility of hallucinated outputs may remain undetected (24). This reinforces the hypothesis that pre-deployment literacy assessment is absent as a standard practice and further suggests that even post-deployment training may be insufficient without structural safeguards that do not depend solely on individual clinician vigilance at the moment of clinical use.

#### 3.2.4 Sociodemographic bias in LLM clinical reasoning

The case elaborated in the above Section 3.1.2, illustrated that structural health inequities can be encoded into algorithmic predictions through the choice of a cost-based proxy variable. Evidence on LLMs suggests that sociodemographic bias is neither eradicated nor diminished in generative AI systems and may be present through many and less detectable mechanisms.

Omar et al. evaluated nine LLMs, both proprietary and open source, across more than 1.7 million outputs generated from 1,000 emergency department cases each presented in 32 sociodemographic variations with clinical information held constant (25). Cases labelled as Black, unhoused, or LGBTQIA+ were more frequently directed toward urgent care, invasive procedures, or mental health assessment compared to those deriving from the reference group. LGBTQIA+ subgroups received mental health assessment recommendations approximately six to seven times more often than other groups. Furthermore, patients with high-income were recommended to receive advanced imaging significantly more often than those from while low and middle-income cases. These patterns were consistent across all nine examined models and remained in place after multiple-hypothesis corrections. In addition, differences in the AI responses lacked validation from clinical reasoning or established guidelines, indicating model-driven bias instead of clinically justifiable variation.

A systematic analysis of 24 studies examining demographic disparities in medical LLMs found that 22 studies (91.7%) detected biases, including gender bias present in 15 of 16 studies (93.7%) and racial or ethnic bias in 10 of 11 studies (90.9%) (26). Only two studies found little or no bias in certain contexts, suggesting that sociodemographic bias in medical LLMs is not an anomaly but a systematically recurrent feature that cuts across model types, sizes, and clinical areas.

When viewed through the literacy lens suggested by the author, this pattern is a more complicated governance issue that is different in quality from the Obermeyer issue. In that case, bias could be technically corrected once it was understood, because it was linked to a specific design choice which was using healthcare costs as a proxy for health need. However, in LLMs, sociodemographic bias is spread out across the model architecture, the training data composition, and the new reasoning patterns that come up, resisting *post-hoc* correction. Governance professionals and clinical procurement teams using LLMs for triage, documentation, or decision support therefore need to know how to recognize bias and how to interpret fairness metrics to understand why bias exists and identify the structural changes needed to fix it.

#### 3.2.5 Continuity with earlier failure patterns

These generative AI findings do not represent an entirely new category of implementation risk. Rather they confirm that the five failure patterns observed in the three predictive AI examples are replicated in a more complex epistemic context. The fluency of LLM outputs raises the scrutiny required before a healthcare or governance professional can safely rely on a recommendation. Many commercial LLM installations are not open to independent review, which is what responsible procurement requires. Furthermore, the lack of approved AI literacy assessment techniques prevents those deploying these systems from determining whether clinicians, patients, or governance professionals engaging with them have the skills to do so in a safe manner. The generative AI evidence reinforces, as opposed to altering the framework suggested in this paper that quantifiable, tier-specific AI literacy competencies are essential for trustworthy AI implementation in the healthcare setting.

## 4 Current measurement tools: the AI literacy gap

### 4.1 Shortcomings of existing tools

The eHEALS, developed in 2006, has eight items assessing respondents' perceived ability to find, evaluate, and use online health resources (8). Its limitations are significant when it comes to assessing AI-specific skills. It is temporally obsolete, having been developed prior to the advent of smartphones, patient portals, wearable health devices, and all contemporary AI applications used in clinical settings. It assesses perceived rather than actual skills: a Dutch validation study demonstrated that eHEALS scores do not predict performance on practical health information tasks and fail to consistently differentiate between individuals with low and high levels of health-related internet proficiency (9). Furthermore, it lacks any components that pertain to algorithmic comprehension, bias identification, probabilistic inference, or data governance.

More recent tools address some of these problems, though none addresses all of them. The Adult Inpatient eHealth Literacy Scale (AIPeHLS) (27) identifies AI-generated health content and hallucinations as emerging digital health literacy issues within a Web 3.0 context, yet it is limited to hospital settings, it relies on self-reporting, and lacks elements of fairness literacy. An adapted version of the eHEALS for social media contexts (28) broadens the instrument's applicability to health information found on social platforms. However, it does not appertain to the clinical AI systems that are the focus of this paper. The Digital Health Literacy Instrument (DHLI) shows broader validity than the eHEALS across a general adult population (29) but was designed to assess Health 1.0 and 2.0 skills, not AI-specific competencies.

### 4.2 Six absent AI literacy dimensions

Following the aforementioned case study analysis and an examination of the most frequently referenced validated digital health literacy measuring tools, this study suggests six competency categories as preliminary objectives for the creation of new instruments. The dimensions are derived from: recorded failure patterns in the AI implementation cases analyzed in Section 3, deficiencies identified in the eHEALS, DHLI, AIPeHLS, and modified eHEALS instruments, and competency frameworks from the responsible AI literature (16). They do not result from any formal systematic audit of instruments and it is recognized that some instruments may partially include certain dimensions. Their psychometric qualities remain untested, and their mutual independence, internal structure, and correlation with observable outcomes necessitate additional empirical examination. They are presented here as a framework for future instrument development.

The measurement theory comment indicates that the suggested framework is based on a competency-based assessment paradigm (30, 31) rather than on a knowledge-recall paradigm. Each characteristic below must be evaluated using performance-based approaches that reflect actual ability rather than perceived self-efficacy. The six aspects are outlined below in a systematic fashion detailing their origin, definition, illustrative performance indicators, and suitable assessment method.

#### 4.2.1 Algorithmic literacy

This dimension identifies a gap visible in both the eHEALS and DHLI, and is supported by work on dataset shift (18). It aligns with the AI trustworthiness framework of the EU HLEG (2019) and the overarching principles of responsible AI (16). At its core, algorithmic proficiency fundamentally involves the ability to understand that AI systems generate probabilistic outputs rather than definitive conclusions, to differentiate correlation from causation, to interpret confidence intervals, and to identify when a model may be operating beyond the population for which it was validated. In practice, this may involve correctly interpreting an AI-generated risk score in light of its accompanying confidence interval or identifying why a model validated in one hospital context cannot be applied to a different patient population without revalidation. Appropriate assessment formats include vignette-based tasks, OSCE stations, and structured clinical evaluations.

#### 4.2.2 Bias awareness

The absence of this feature in eHEALS and DHLI is well-supported by empirical research, particularly (4) analysis of racial bias in healthcare algorithms and the findings concerning sociodemographic discrepancies in clinical AI (25). It is also directly relevant to fairness requirements under Article 13 of the EU AI Act. The competency involves recognizing that AI systems can embed and amplify pre-existing structural inequalities, identifying performance variation across demographic subgroups, and applying fairness metrics including demographic parity, equalized odds, and calibration to evaluate AI recommendations critically, especially when a patient's profile diverges from the model's training population. An illustrative performance indicator would involve acknowledging that using healthcare expenditure as a surrogate for health necessity will consistently undervalue the needs of populations historically marginalized from care, or, precisely analyzing a fairness audit that reveals varying sensitivities among racial subgroups. Assessment methods include structured document analysis, governance simulation exercises, and vignette-based evaluation.

#### 4.2.3 Comprehension of data governance

Neither eHEALS nor DHLI sufficiently address data governance, despite its increasing significance in the implementation of clinical AI. The justification for its inclusion is based on the analysis of GDPR fragmentation (8), the literature on EHDS governance (32), and the SACHRP rules regarding AI research oversight (21). Practitioners must comprehend how AI systems utilizing patient data affect both individual and group privacy, differentiate between data access and data transmission in federated architectures, and recognize the limitations of de-identification and the dangers of re-identification. They must possess a practical understanding of privacy-preserving methodologies, including federated learning and differential privacy, along with the ability to implement frameworks such as GDPR, EHDS, and HIPAA in particular data processing scenarios. A practitioner exhibiting this ability may precisely identify the relevant GDPR legal basis for a specific AI training scenario and elucidate why permission is not a suitable foundation in that situation. Evaluation may encompass systematic document examination, scenario-driven written assignments, or governance simulation exercises.

#### 4.2.4 Critical evaluation of AI outputs

This dimension, based on research on hallucination in clinical documentation (22) and discoveries on antagonistic hallucination (24), fills a void in both eHEALS and DHLI, while conforming to the human supervision stipulations of Article 14 of the EU AI Act. The competency involves the capacity to differentiate between AI-generated and human-authored content, recognize plausible yet factually incorrect outputs, evaluate the quality and origin of AI-cited sources, and ascertain the contextual appropriateness of AI-generated recommendations prior to implementation. A practitioner should identify a minimum of two clinically significant hallucinations in an AI-generated clinical note and explain their potential implications for patient management. Assessment formats may encompass annotated document analysis, Objective Structured Clinical Examination (OSCE) stations with AI-generated clinical documentation, or predefined patient scenarios.

#### 4.2.5 Calibration of trust

This feature is firmly grounded in empirical research within the human factors' literature, notably the work about automation bias and aligns with the oversight stipulations of Article 14 of the EU AI Act (33, 34). Its omission from eHEALS and DHLI is significant considering its direct impact on patient safety. The competency pertains to the capacity to adjust dependence on AI outputs in a manner that is attuned to task risk, model attributes, and contextual ambiguity. This entails understanding when a recommendation necessitates independent human verification prior to execution, as well as acknowledging the dangers of both excessive dependence and insufficient utilization. In a critical situation, such as an AI sepsis alert triggering at low confidence amid substantial workload, a practitioner exhibiting this competency would acknowledge the necessity for an independent clinical evaluation prior to either responding to or disregarding the alert. Evaluation may encompass simulation exercises, clinical vignette sequences, or think-aloud protocols during AI-assisted activities.

#### 4.2.6 Interpretation of explainability

The final dimension incorporates Article 13 transparency mandates of the EU AI Act, EU HLEG explainability criteria, and a corpus of empirical literature illustrating the challenges faced by physicians and patients in comprehending AI explanations. The competency encompasses comprehending the implications of different explanation types, saliency maps, SHAP values, attention weights, and natural language rationales regarding what they reveal and conceal about a model's reasoning, acknowledging the limitations associated with *post-hoc* explainability methods, and converting technical AI explanations into clinically or personally relevant language for decision-making. A practitioner may illustrate this by accurately interpreting a saliency map in conjunction with a radiology report while also recognizing a methodological limitation of the approach, or by conveying an AI-generated risk explanation in terms comprehensible to a patient without misrepresenting or exaggerating the output. Annotated picture analysis, patient interaction simulations, and written explanation tasks represent viable evaluation formats. Table 2 depicts the six absent health literacy dimensions.

**Table 2:** Six absent AI health literacy dimensions.

Dimension	Derivation sources	Assessment format
1. Algorithmic literacy	eHEALS/DHLI gaps; Finlayson et al. (2021); EU HLEG	Vignette-based; OSCE; structured clinical exam
2. Bias awareness	eHEALS/DHLI gaps; Obermeyer (2019); Omar (2025)	Vignette; document analysis; governance simulation
3. Data governance	eHEALS/DHLI gaps; GDPR fragmentation (11); SACHRP (27)	Document analysis; governance simulation; written exam
4. Critical appraisal of AI outputs	eHEALS/DHLI gaps; Asgari (2025); Omar (2025); AI Act Art.14	Annotated review; OSCE; standardized patient scenario
5. Trust calibration	eHEALS/DHLI gaps; Parasuraman & Riley (1997); Skitka et al. (1999)	Simulation; vignette series; think-aloud protocol
6. Explainability interpretation	eHEALS/DHLI gaps; AI Act Art.13; EU HLEG (25)	Image interpretation; patient communication simulation

### 4.3 The measurement crisis

A recent systematic review of research on AI techniques and health literacy, revealed a measurement gap, identifying 1,296 potentially eligible studies, yet found only 18 (1.4%) met inclusion criteria, with merely 5 involving direct participant engagement (35). In other words, serious empirical research on AI health literacy remains scarce. Pre-deployment literacy assessment is unfeasible without validated instruments, post deployment evaluation fails to identify the difference between literacy-related failures and technical ones and the efficacy of any intervention aimed at enhancing literacy cannot be subjected to rigorous testing. This lack of peer-reviewed empirical research on AI health literacy is also reflected by the small number of references in this paper. This is not an oversight; it is a direct result of a field that remains in nascent phase by any bibliometric criterion. The existing literature is predominantly focused on 2024–2025, underscoring that it is only recently that the scientific community has begun to engage seriously with these inquiries.

## 5 Proposed framework: three-tier AI literacy structure

### 5.1 Framework overview

The framework suggests that literacy is not just an add-on to AI deployment but rather its necessary foundation. It has three levels, each targeting a different dimension of the implementation problem.

**Tier 1 Assessment Infrastructure:** The development of validated, performance-based tools that measure real and not perceived AI literacy, is foundational to the framework. These tools must be sector-specific with distinct assessment tools for clinicians, patients, and governance professionals. Every AI deployment should be preceded by a baseline literacy profile of the relevant stakeholder groups, with longitudinal tracking to monitor the development of AI literacy skills over time.

**Tier 2 Educational Ecosystem:** AI literacy competencies should be integrated into existing training pathways such as academic curricula, ongoing medical education, *ad hoc* patient education and training programs. Furthermore, interdisciplinary programs bridging clinical, technical and ethical content are important to break the silos that current AI fragmented governance has created. Finally, community based grassroot initiatives can prevent digital divides and ensure that populations at greater risk of exclusion from AI-mediated care, are not left behind.

**Tier 3 Governance Integration:** AI deployment authorization should require a structured literacy impact assessment as a prerequisite, while institutional quality frameworks should incorporate literacy measurements alongside clinical outcome indicators.

Table 3 depicts the three tier AI health Literacy Framework: Competencies, Assessment, Strategies and Responsible actors.

**Table 3:** The three-tier AI health literacy framework: competencies, assessment strategies, and responsible actors.

Tier	Focus	Target stakeholders	Core competencies	Assessment strategy	Responsible actors
Tier 1 Assessment Infrastructure	Measuring demonstrated AI literacy before and during deployment	Clinicians, patients, governance professionals	Baseline competency profiling across all six AI literacy dimensions; longitudinal tracking of literacy development	Validated, performance-based instruments distinct from self-report tools; sector-specific assessments calibrated to system risk level	Healthcare institutions; accreditation bodies; AI developers; regulators
Tier 2 Educational Ecosystem	Building AI literacy competencies across training pathways	Medical students, residents, practicing clinicians, patients, community health workers	Algorithmic reasoning; bias recognition; trust calibration; explainability interpretation; data governance comprehension; critical appraisal of AI outputs	Integration into medical school curricula and CME; patient portal-embedded just-in-time learning; community-based digital literacy programs	Medical schools; professional bodies; patient advocacy organizations; public health agencies
Tier 3 Governance Integration	Embedding AI literacy as a governance prerequisite	Privacy officers, IRB members, procurement teams, institutional administrators, policy makers	Fairness metric interpretation; privacy-preserving architecture comprehension; regulatory mapping; vendor claim evaluation; cross-disciplinary translation	Literacy impact assessments as deployment precondition; demonstrated competency standards for governance committee membership;	Regulatory agencies; ethics committees; health ministries; institutional leadership

### 5.2 Sector-specific competency maps

#### 5.2.1 Clinicians

Clinician AI literacy includes four overlapping areas: technical competency (being able to read validation metrics and understand calibration and generalizability), clinical application (putting AI into practice workflow, dealing with alert fatigue, communicating about AI with patients), ethical reasoning (checking for fairness, recognizing bias, understanding accountability frameworks), and governance engagement (evaluating vendors, setting procurement standards, participating in quality monitoring). Medical school curricula, evidence-based medicine training, residency and fellowship programs with specialty-specific AI content, and hospital credentialing requirements for AI-assisted clinical specialties represent appropriate integration points for these skills.

#### 5.2.2 Patients

For patients, the most important skills include being able to read AI-flagged results in patient portals, knowing what probabilistic risk scores mean and do not mean, knowing when AI outputs need to be checked by a person before acting on them, being able to use AI-mediated care pathways such as chatbot triage and automated scheduling, and understanding how their data might be used in AI training. Explainability and health literacy-responsive design should be two guiding design principles for AI systems used by patients. AI outputs should not merely be shown to patients, but they should also be explained through layered, user-controlled educational content that is delivered at the moment the output is seen.

An equity imperative runs through all aspects of patient literacy programming. Interventions must be structured to mitigate known disparities in digital health literacy across lines of age, gender, race, education, language, and socioeconomic status. Evidence demonstrates that these disparities are associated with health-related behaviors independently of other factors (19, 36).

#### 5.2.3 Communities and public health

Community health workers are especially important as literacy intermediaries for underserved populations. The digital training programs that have helped bring telehealth access to these communities need to be expanded to include AI-specific skills (36). The core competencies required include understanding the limitations of AI symptom checkers and chatbots, knowing when and how to question algorithmic recommendations, explaining AI-generated health guidance in simple terms, and advocating for community needs in AI design and procurement processes. The author's recommendation is that AI tools meant to be used by or with community health workers should be developed in collaboration with those workers from the earliest stages, rather than developed independently and subsequently introduced through training. This principle is well established in the participatory design literature, though its application specifically to AI health literacy programs remains to be empirically tested.

#### 5.2.4 Governance in professionals

For AI governance to be effective, those in charge privacy officers, IRB members and institutional administrators must have the technical literacy to deal with AI-specific risks. However, governance roles in healthcare are rarely defined with any AI competency requirements in mind (37). There are three main areas of knowledge that are needed: 1) a technical understanding of privacy-preserving architectures and their known attack vectors 2) the ability to map regulatory requirements (e.g., GDPR for Europe or HIPAA for the US) onto their technical safeguard equivalents and to see where technical compliance leaves gaps in protection and 3) the ability to coordinate across disciplines, such as facilitating productive conversations between data scientists, clinicians, lawyers, and ethicists, and translating technical documentation into language that non-specialist decision-makers can understand. Furthermore, foundational AI literacy training and competency assessment should be prerequisites for the exercise of approval authority, not optional supplements to it, and governance committee composition should require demonstrated competency across clinical, technical, ethical, legal, patient advocacy, and data governance domains.

## 6 The European lesson: harmonization and fragmentation

### 6.1 GDPR

The GDPR was designed to harmonize data protection across European member states, and for researchers seeking to use health data for secondary purposes, it initially held the promise of reduced administrative complexity. Instead, however, it produced the fragmentation it was meant to prevent (38). Biobanks operating under similar rules began to adopt very different ways of obtaining consent from the participants, ethics committees turned data access into procedural hurdles, and data protection officers demanded multiple layers of authorizations all practices resulting from the misinterpretation of the legal basis for health data processing (Article 6(1) read in conjunction with Article 9(2)(j) and Article 89(1) GDPR). As a result, rather than cutting through red tape, GDPR created different approval processes for each institution and even more divergent ones for cross border collaborations (6). The root cause was not a deficiency in the legal text *per se*, but a deficit in the critical thinking needed to apply that letter of the law consistently and correctly.

However, it would be a mistake, to treat this interpretive deficit as the sole reason for fragmentation. The Regulation itself created substantial room for divergence, and several of its features rendered fragmentation more than probable. More specifically, health research was deliberately left incomplete with Article 9(4) expressly permitting Member States to maintain or introduce further conditions for the processing of health and genetic data and Article 89(2)(3) leaving the design of research safeguards and derogations largely to national law. The provisions governing secondary use of health data were thus drafted to be specified differently across the various Members States jurisdictions. This latitude was exercised by national legislators that were already far from being uniform, as Member States entered the “GDPR era” with research ethics infrastructures, informed consent procedures, and institutional cultures that had developed very differently over the previews decades. Two further factors reinforced this trajectory. First, authoritative interpretive guidance from the European Data Protection Board and national supervisory authorities emerged slowly, and often only after institutions had already committed to a particular approach. Second, with substantial fines attached to non-compliance, institutions had every reason to default to the most restrictive interpretation of the letter of law.

Set against this background, the literacy deficit identified here is best read as a complement to the structural explanation rather than a rival to it, one largely overlooked cause of fragmentation among several others. What it adds is an account of the variation that the structural factors leave unexplained. National law and institutional frameworks may explain why practices diverged from one jurisdiction to another, but they cannot explain a more puzzling pattern: institutions bound by the same provisions, operating within a single legal system and facing broadly similar conditions, still interpreting those provisions in entirely different ways. When the structural explanations have been accounted for and that kind of divergence remains, the explanation must lie somewhere else, in the differing capacity of the people charged with interpreting and applying the framework.

### 6.2 Implications for the European health data space

The EHDS (Regulation (EU) 2025/327, which entered into force on March 26, 2025, with a phased applicability structure distinct from its entry into force: the European Commission must adopt key implementing acts by March 2027. Primary use provisions and secondary use rules for most data categories become applicable in March 2029 and the second group of primary data categories, including medical imaging, laboratory results, and hospital discharge reports, become applicable across all Member States in March 2031) represents the next attempt that European Union is making to overcome this misinterpretation of the law through standardized data governance, Health Data Access Bodies, technical interoperability for cross-border secondary use, and privacy-preserving computational architectures. The risk, however, is that the GDPR pattern will repeat itself. Legal harmonization without corresponding health governance literacy harmonization will once again produce fragmented, inconsistent implementation (6, 22). A study of expert interviews focused on EHDS implementation and revealed that the techno-legal dimensions of balancing data reuse and protection are not adequately comprehended, highlighting regulatory fragmentation as a significant governance risk which needs harmonized policies and more clear legal definitions among EU Member States (39). Similarly, an independent assessment of the EHDS has flagged that the Regulation carries the significant risk of intensifying digital disparities for those who may lack the digital literacy or resources necessary to engage with and benefit from the EHDS (40, 41).

### 6.3 Lessons for broader AI governance

The EU Artificial Intelligence Act (Regulation (EU) 2024/1689), effective since August 1, 2024, transforms AI literacy from a recommended best practice to a legal requirement (42). Article 4 of the AI Act, effective since February 2, 2025, mandates that providers and deployers of AI systems take measures to ensure a sufficient level of AI literacy among their staff and other persons dealing with the operation and use of those systems. Article 3(56) defines AI literacy as the skills, knowledge, and understanding that enable providers, deployers, and affected persons to make informed deployment decisions and to recognize the opportunities, risks, and potential harms associated with AI. As a matter of current EU law, this obligation encompasses all risk categories, not only high-risk systems, and its early application, alongside the prohibition of certain AI practices, indicates that the European legislator views literacy as fundamental rather than ancillary to AI governance (39, 42).

The AI Act enhances literacy obligations by instituting transparency and human oversight requirements for high-risk AI systems. AI Act Article 13 mandates that high-risk AI systems must be engineered to guarantee operational transparency and that deployers must provide instructions that are succinct, comprehensive, and easily understandable. Article 14 mandates that high-risk AI systems, including those deployed in the healthcare space, must ensure human oversight (42). Furthermore, Article 14 makes clear that human monitoring is not only a structural necessity but also a competency prerequisite. Supervision devoid of literacy is not genuine supervision.

The European Commission's guidance on Article 4 recognizes the structural challenges posed by the literacy duty. As currently framed, Article 4 does not mandate the assessment of staff knowledge, nor does it define what constitutes an adequate level of AI literacy, acknowledging the necessity for adaptability given the swift evolution of technology and its applications (39). The Commission has identified three essential compliance orientations: organizations must cultivate a fundamental comprehension of AI, assess their role as either provider or deployer, and tailor employee knowledge to the risk level of the AI systems they engage with (39). This flexibility is necessary given the pace of technological change. However, it also engenders the specific risk of fragmentation, exemplified by the GDPR experience, whereby when legal obligations are broad and inadequately defined, their execution will differ based on the interpretive capacity of those tasked with implementing them.

This governance challenge is further compounded by the broader EU digital health regulatory architecture within which AI systems in healthcare operate. The European Health Data Space (Regulation (EU) 2025/327), published in the Official Journal of the European Union on March 5, 2025 and entering into force on March 26, 2025, establishes the regulatory framework governing the access, exchange, and secondary use of electronic health data across the EU. The Regulation operates on a distinct three-stage applicability structure. First, the European Commission is required to adopt key implementing acts, which will define the operational rules and technical specifications necessary for the Regulation to function in practice, by March 26, 2027. Second, on March 26, 2029, the first substantive provisions become applicable: primary use obligations including the cross-border exchange of patient summaries and ePrescriptions, and secondary use rules for most electronic health record data categories. Third, on March 26, 2031, the second group of primary data categories, encompassing medical imaging, laboratory results, and hospital discharge reports, will become applicable across all Member States, alongside secondary use provisions for remaining data categories such as genomic data (38). AI literacy is therefore not only an AI Act compliance matter but a prerequisite for meaningful engagement with the EHDS framework, as clinicians, administrators, and data stewards must be equipped to navigate both regulatory regimes concurrently. It should be noted that the AI Act does not currently require validated literacy assessments, define minimum competency thresholds, or mandate the specific tiered framework proposed in this paper; these are the author's governance recommendations, informed by the Act's stated purposes.

Beyond what the AI Act currently requires, this paper recommends addressing the significant circular governance issue that the current framework creates. The literacy requirements of the AI Act will be executed by organizations whose own AI literacy levels determine whether those duties are understood and fulfilled meaningfully rather than merely procedurally. Healthcare organizations implementing AI systems categorized as high-risk under the AI Act, which encompasses AI systems utilized in health and life sciences as outlined in Annex III, must ensure that clinicians and administrators designated for human oversight roles possess the requisite competencies for those positions. As a matter of institutional design, the author proposes that without validated tools to assess AI literacy, established competency standards, and the training infrastructure proposed in this paper, compliance with Articles 4, 13, and 14 risks deteriorating into the governance theater described in Section 6.1 (39, 42). The phased application of the EHDS through 2031 offers a structured window of opportunity to embed such literacy infrastructure before full regulatory enforcement takes effect, but only if preparatory action begins now.

## 7 Implementation framework

### 7.1 Medical education integration

The American Medical Association, during its November 2025 Interim Meeting, recognized that current medical training inadequately equips physicians for practice in an AI-integrated clinical settings and identified an urgent need for a standardized AI literacy framework throughout the medical education continuum (43). The proposed integration commences in the pre-clinical years, incorporating fundamental AI concepts into current biostatistics and epidemiology curricula, fostering critical appraisal skills specifically for assessing AI studies, introducing the principles of algorithmic bias and their health equity implications, and establishing foundational data governance literacy. During clinical rotations, students should experience specialty-specific AI applications and their limitations, acquire practical experience with clinical decision support systems, cultivate the communication skills needed to discuss AI outputs with patients, and analyze real-life case studies. Residency and fellowship training must cultivate the advanced competencies necessary for AI-intensive specialties, instruct physicians in the local validation of deployed AI tools, and build the capacity for significant involvement in hospital AI governance.

Assessment methodologies must evolve beyond knowledge recall toward evaluation of demonstrated competency. These include OSCE stations necessitating students to interpret AI outputs in simulated clinical scenarios, written examinations assessing understanding of validation metrics, and portfolio assessments in which trainees document and reflect on AI-related clinical decisions. Accreditation organizations should convert these priorities into clear AI literacy indicators within competency milestone frameworks.

### 7.2 Designing patient education

The principle of layered progressive disclosure should guide the design of patient portal-based education. By default, there should be a brief, easily comprehensible summary of any AI output with the option to expand into more detailed explanations and, for those who want it, technical background, all of which should be user-controlled and available when the AI output is encountered. When new AI features are introduced, there should be interactive tutorials with built-in feedback. Content should be able to change based on the person's reading level and how they prefer to study. Community-based programs must specifically focus on populations most vulnerable to be marginalized by AI-mediated care like older adults, individuals with limited health literacy, and rural and underserved communities. These programs should be implemented through trusted intermediaries including community health workers, public libraries, and established community organizations. Community health workers, who are important facilitators of mobile health access for underserved populations (44), could also have their existing training extended to add AI literacy competencies.

### 7.3 Literacy impact assessment for AI deployment

Healthcare organizations must perform a systematic literacy effect evaluation prior to implementing any AI system for clinical or operational purposes. The five-step procedure detailed below serves as a comprehensive foundation. However, a risk-stratified approach is more suitable in practice. A comprehensive assessment is necessary for systems deemed high-risk, such as those categorized under EU AI Act Annex III or similar national regulations, whereas lower-risk tools may necessitate less rigorous alternatives, like a focused competency gap analysis aimed at the primary operator group. This progressive approach embodies the concepts of proportionality inherent in both EU and US regulatory frameworks and is far more practicable given the resource limitations encountered by most healthcare organizations.

The initial phase involves stakeholder mapping and more specifically recognizing the entities that will be impacted by the system or hold accountability for its utilization and oversight. The second component is baseline assessment, which entails the administration of validated AI literacy tools to each stakeholder group to ascertain current skill levels and identify deficiencies in relation to the demands of the system on its users. The third phase involves focused intervention creating training programs and decision-support resources that specifically address the identified shortcomings, rather than implementing generic digital literacy initiatives that may not be tailored to the system in question. The fourth is the formulation of deployment criteria, specifically, delineated minimum literacy standards, that must be achieved prior to the authorization of independent usage. The fifth and last step is continuous evaluation by integrating monitoring mechanisms to assess the progression of literacy levels over time and their correlation with AI-related results in practice.

The principal practical obstacles to executing this type of examination are readily foreseeable. Resource limitations regarding time, personnel, and finances are frequently mentioned and exacerbated by the lack of validated instruments a concern which is also directly addressed in the research agenda outlined in Section 9, along with the organizational challenge of incorporating a new assessment process into procurement and deployment workflows that already bear a considerable administrative burden. The most efficacious approach to these obstacles is integration rather than supplementation. Literacy assessment must be integrated into the current clinical system procurement process, executed concurrently with technical validation instead of sequentially afterward, with results contributing to existing governance committee evaluations rather than creating a distinct institutional procedure. Organizations that have implemented structured pre-deployment checklists, such as the NHS Digital Technology Assessment Criteria (DTACs), are optimally situated to integrate a literacy impact assessment module into their existing frameworks with minimal additional burden.

### 7.4 Regulatory and policy levers

Before any AI system can be used, accreditation criteria should involve a written literacy evaluation. They should also require ongoing monitoring of AI-related adverse events that might occur and a systematic analysis of whether literacy deficits contributed to the generation of these events or not. Professional licensure frameworks might also include AI literacy requirements in continuing medical education and, for specialties which use a lot of AI (e.g., radiology) in competency standards specific to those specialties. The current regulatory pathway for AI as medical device software evaluates technical performance but does not consider whether the potential users can safely interpret or act upon the outputs the system produces. Requiring user comprehension studies as part of pre-market approval and mandating AI literacy labeling (specifying the minimum competencies required for safe independent use), would be a solid first step to address this important gap. Research funding organizations should set up separate ways for AI literacy research and require that AI implementation studies include literacy assessment as a component of their evaluation methodology.

## 8 Discussion

### 8.1 The connection between literacy and responsible AI frameworks

The responsible AI literature has converged on several fundamental principles: fairness and non-discrimination, transparency and explainability, accountability and governance, privacy and security, and safety and robustness. The framework proposed in this paper aligns with recognized implementation science paradigms. The Normalization Process Theory delineates sense-making (cognitively embedding the work), enrollment (getting people to commit to it), and appraisal (reflexively monitoring it) as critical normalization mechanisms (40). AI health literacy, as proposed here, is precisely the cognitive and evaluative infrastructure that enables each of these mechanisms to function at the level of interacting with AI systems. The Consolidated Framework for Implementation Research similarly highlights elements of the inner setting such as implementation climate and readiness for implementation as predictors of successful adoption. Literacy assessment provides the empirical basis for evaluating readiness (45). Research on human variables regarding automation bias (33, 34) provides direct empirical support for the trust calibration dimension, while extensive experimental and field evidence shows that users over-trust automated recommendations when the cognitive tools to appraise them are absent, a pattern consistent with the failure modes identified in the case studies. In the context of patient safety, literacy deficiencies of the kind outlined in this paper are best understood as pre-existing vulnerabilities in the system that do not cause failures directly but increase the probability that active errors will propagate rather than be caught (46).

### 8.2 International applicability

The legislative analysis in this article is primarily based on European frameworks, GDPR, EHDS, and the EU AI Act, although neither the primary argument nor the three-tier system it underpins should be perceived as geographically particular. The literacy gap identified in this paper is not a byproduct of European regulatory framework. It represents a structural issue that emerges whenever AI systems are implemented in healthcare environments more rapidly than institutions can cultivate the necessary human competency infrastructure for their responsible use and governance.

This trend is evidently observable outside of Europe. In the United States, the FDA's regulatory framework for AI/ML-based Software as a Medical Device assesses technical performance and safety attributes but it does not consider user understanding as a prerequisite for implementation. SACHRP (2022) has officially recognized deficiencies in governance literacy concerning AI research supervision, and the ONC's execution of the 21st Century Cures Act has not yet integrated AI literacy mandates into its interoperability or clinical decision support frameworks. Collectively, these deficiencies indicate that the issue this research examines, although manifested differently, exists in the American regulatory framework as well as in the European context.

This is also applicable at the global level. The WHO's framework on the ethics and governance of artificial intelligence for health (47) regards health workforce capacity, especially the essential literacy to securely deploy and supervise AI tools, as a fundamental prerequisite for implementation rather than a secondary issue. In low- and middle-income nations, the difficulty is exacerbated as AI health literacy cannot be established on a digital literacy framework that is itself inadequate, and the equity ramifications of that disparity are correspondingly more acute. Adaptations that consider varying resource contexts, institutional structures, and population-level digital literacy will be crucial for the practical use of any paradigm derived from this study in such environments.

The proposed framework is presented as a generalizable scaffold rather than a jurisdiction-specific tool. The fundamental premise, that a systematic disparity exists between the technical competence of implemented AI systems and the expertise of people overseeing them permeates healthcare institutions and regulatory frameworks. The precise regulatory integration points, pertinent institutional players, and the adjustment of competency criteria will necessitate local adaptation to the healthcare system architecture and legislative frameworks in which implementation takes place.

### 8.3 Why literacy has been overlooked

Several structural factors help explain why literacy has received such little attention in responsible AI discourse. Disciplinary silos are part of the answer: computer scientists, ethicists, legal scholars, and health literacy researchers rarely read the same journals or attend the same conferences, and the cross-pollination that might have brought literacy into the responsible AI conversation has not occurred on a wide scale. Technical solutionism is another factor: there is a persistent tendency to assume that well-designed and adequately explainable algorithms will eventually render user understanding unnecessary, a belief that the evidence does not support. The measurement gap reinforces the neglect: without validated instruments, literacy deficits are invisible in the data, and what cannot be measured tends to not be managed. Additionally, there are institutional incentives to avoid confronting literacy deficits directly: acknowledging that current deployment is proceeding on a foundation of inadequate stakeholder competency creates uncomfortable liability exposure and challenges professional self-perceptions in ways that organizations and professions are understandably reluctant to invite.

This paper does not contend that literacy deficits are the exclusive or principal explanation of AI deployment failures in healthcare. Implementation science and organizational theory offer comprehensive and well-validated explanations for the reasons AI systems fail, which go well beyond individual competency gaps. Technical factors, such as insufficient model generalizability, inadequate external validation, dataset shift, and architectural limitations that resist *post-hoc* debiasing, are well-documented in the literature as potential proximate causes in contexts such as those reviewed here. Organizational factors, including misaligned financial incentives for rapid procurement, vendor capture, cultural resistance to algorithmic oversight, and the structural fragmentation of governance responsibility across clinical, technical, legal, and administrative silos, represent additional systemic contributors. Economic factors, including the competitive pressure to deploy AI systems before independent validation is complete and the commercial opacity of proprietary systems, further compound the governance challenge. The argument advanced here is not that literacy gaps caused these failures but rather that literacy gaps represent one significant, under-investigated, and actionable contributing factor that has been systematically overlooked because it is difficult to measure. Literacy gaps also create institutional discomfort when acknowledged and lack the validated assessment infrastructure that other governance factors enjoy. The literacy framework proposed here is intended to complement, not replace, the technical, organizational, and economic explanations that the responsible AI literature has already developed.

### 8.4 From training problem to infrastructure requirement

The central reorientation this paper calls for is a fundamental shift from perceiving literacy as a training problem to recognizing it as an essential infrastructure requirement. Currently, healthcare organizations treat AI literacy as a secondary consideration, a one-time induction module delivered after deployment focused on procedural steps and recorded as complete when attendance is logged. The proposed alternative positions literacy as a prerequisite rather than an adjunct, which is systematically evaluated before deployment, is continuously cultivated through incorporation into professional training pathways, and is measured not by completion rates but by exhibited proficiency in AI-related decision-making and evidence of suitably calibrated trust in AI outputs.

### 8.5 The trust cascade

Trustworthy AI is often framed primarily as a technological achievement assuming that if systems are demonstrably fair, transparent, and accountable, then trust will automatically follow. This framing distorts the true relationship. In practice, trust arises from AI health literacy. Users can appropriately calibrate their faith in an AI system/tool and neither mindlessly defer nor dismiss it without engagement when they comprehend its functions, underlying assumptions, and potential shortcomings. Transparency features are intended for audiences capable of interpreting them, which allows explainability to foster authentic comprehension rather than merely providing an illusion of openness over a black box. In the absence of literacy, this sequence deteriorates. When AI health literacy is absent, users tend toward one of two failure modes: either uncritical dependence on AI outputs or outright rejection of them. Either path leads to misuse and erodes the user confidence and trust that the AI systems require to be clinically beneficial.

### 8.6 Literacy and health equity

Van Kessel and colleagues have proposed that digital health literacy may function as a super-determinant of health, a factor with the potential to shape access to and use of a broad range of health-related resources and services across multiple outcome domains, while explicitly acknowledging that the empirical evidence for this relationship remains incomplete and uncertain (19). Whether or not this framing is ultimately vindicated by the evidence, the equity implications of AI deployment are difficult to dispute. Patients with limited digital literacy face genuine barriers to navigating AI-mediated care pathways. Communities without adequate digital infrastructure are structurally excluded from the benefits of AI-enhanced care. Clinicians serving historically underserved populations may have had less exposure to AI literacy training. Governance bodies that lack diversity of representation make decisions that may systematically fail to account for the needs and vulnerabilities of marginalized communities. These dynamics are not hypothetical: they are the predictable consequences of deploying AI without equity-conscious literacy infrastructure.

These disparities are not spread evenly. They gather among those groups of people and their communities, which are already under served. Older adults consistently score lower on digital health literacy than younger ones. People with less formal education or fewer economic resources struggle not just to reach AI-mediated information but also to judge it critically once they have accessed it. Furthermore, linguistic minorities are often handed AI tools that have been built, and tested mainly in dominant languages, while people with disabilities often meet interfaces that where not designed with their special needs in mind. Rural communities carry a double burden with patchy digital infrastructure and less exposure to AI-supported care to begin with, while in low- and middle-income settings the AI literacy gap is layered over more basic shortfalls in basic digital and health literacy. The common thread is that AI health literacy cannot be understood, or improved, by looking at a population average, because the average is precisely what conceals the gaps that matter most. What is needed instead is stratified measurement, so that shortfalls can be located, tracked, and addressed within the specific groups where they run.

Conversely, with deliberate and well-resourced literacy initiatives designed specifically to reach historically excluded groups, AI has the potential to become a tool for democratizing clinical expertise rather than concentrating it further.

### 8.7 Limitations

This paper has certain limitations that should be acknowledged. It is a conceptual framework paper that synthesizes existing evidence; it does not present primary empirical data, and its central propositions are therefore hypotheses rather than findings. The three-tier architecture proposed requires empirical validation before it can be used as the basis for policy or practice. The case study analyses draw on publicly available accounts of AI implementation difficulties, which may not fully capture the institutional complexity of each situation, and which may be subject to reporting bias. The six proposed AI literacy dimensions and the competency maps for each stakeholder group reflect the author's synthesis rather than the output of a validated instrument development process; the feasibility of measuring these dimensions and their psychometric properties remain to be tested. The pace of AI development may also outstrip the capacity of any static literacy framework to remain relevant. Finally, the implementation barriers are real and substantial: resource constraints, professional cultures resistant to acknowledging competency gaps, siloed institutional structures, and misaligned financial incentives may all impede the translation of this framework into practice.

An additional limitation of the narrative review methodology is its vulnerability to selection bias: despite the clear theoretical framework directing source selection, it remains possible that the evidence compiled here aligns more closely with the author's preconceived hypotheses than with the comprehensive spectrum of available viewpoints.

### 8.8 Research agenda

Future research should rigorously examine the framework's core assertions, including the potential correlation between literacy disparities and implementation difficulties through a systematic empirical investigation utilizing prospective study methodologies.

The framework suggested in this paper will remain a theoretical contribution until it is empirically validated. Future research should rigorously scrutinize the framework's core assertions, formostly the plausible relationship between literacy disparities and implementation failures, through systematic empirical investigation utilizing prospective study designs. It should also test the adaptive mechanisms discussed in Section 7 that any literacy framework will require to remain aligned with the pace of AI development. A phased method to validation is proposed in the following research pathway.

#### 8.8.1 Phase 1

##### 8.8.1.1 Expert consensus and dimension validation (years 1–2)

A multi-round Delphi study engaging AI researchers, clinical informaticists, health literacy scholars, patient advocates, governance professionals, and regulatory experts to establish consensus on the six proposed AI health literacy dimensions, their definitions, and their relative priority for each stakeholder group. The Delphi procedure must be conducted by an expert review panel with patient and community representative participation to ensure equity-conscious dimension specification.

#### 8.8.2 Phase 2

##### 8.8.2.1 Instrument development and cognitive interviewing (years 1–3)

Creation of performance-based assessment items for each dimension and each stakeholder tier, succeeded by cognitive interviewing with samples from the target populations (clinicians, patients, governance professionals) to evaluate item comprehension, cultural appropriateness, and discriminant validity. Items must be developed simultaneously in multiple languages due to the international scope of the governance challenge.

#### 8.8.3 Phase 3

##### 8.8.3.1 Psychometric validation (years 2–4)

Pilot conducting and confirmatory analysis of the proposed instruments with adequately powered and purposively diverse samples assessing internal consistency, test–retest reliability, construct validity (convergent and discriminant), and criterion validity when suitable outcome measures are identifiable. Structural equation modelling should be employed to evaluate the hierarchical assumptions of the AI literacy stack model.

#### 8.8.4 Phase 4

##### 8.8.4.1 Implementation pilot studies (years 3–5)

Future longitudinal studies will incorporate literacy assessments into the implementation of AI at partner institutions and track correlations between baseline literacy profiles, training interventions, and clinical outcomes related to AI (including adverse events, alert fatigue, failure to escalate, and documentation accuracy). This research will produce the first empirical data on whether literacy-as-infrastructure, as described by the three-tier paradigm, is a predictor of safer and more equitable outcomes of AI implementation. Priority should be given to Phases 1 and 2 since the approval of assessment tools is the key barrier to the installation of any other framework components. Validated instruments for AI health literacy, should be positioned as a key research investment for public health systems from funding agencies, accrediting bodies, and AI regulatory authorities.

## 9 Conclusion

This paper has argued that AI failures in healthcare may be understood, at least in part, through a literacy lens that has been insufficiently explored in existing implementation and governance research. The case studies analyzed here reveal a recurring pattern in which the stakeholders responsible for deploying, using, and governing AI systems appear to have lacked competencies that could have prevented the failure, though the causal contribution of these potential gaps cannot be established from the available evidence and require empirical investigation. Technical sophistication in the absence of human comprehension produces what this paper calls governance theater.

Existing digital health literacy frameworks are inadequate to address this issue. The eHEALS tool was not designed to assess algorithmic literacy, bias awareness, data governance understanding, critical appraisal of AI outputs, trust calibration, or explainability interpretation. No validated instrument currently fills this gap, while AI systems keep being deployed at scale in its absence.

The reconceptualization this paper calls for is conceptually straightforward but demanding on an institutional level. It calls for AI health literacy to be treated as infrastructure rather than merely training. When it comes to healthcare, AI health literacy shapes whether fairness audits detect real bias, whether explainability features enable understanding, whether accountability structures produce genuine responsibility, whether consent reflects authentic autonomy, and whether safety mechanisms function as designed in the clinical setting.

The research agenda this framework requires is substantial. Validated, sector-specific literacy assessment tools must be developed and tested. Prevalence studies are needed to quantify where the gaps are largest. Over time, literacy measurement must be woven into accreditation, quality measurement, and regulatory frameworks, not as mere add-ons, but as the foundation on which trustworthy AI deployment is built. One thing must remain clear, however. The three-tier framework offered here is a hypothesis and not a tested model. Its central claims that AI health literacy gaps and implementation failures are linked, and that the competencies it describes really do build on one another in the order proposed here, still needs empirical verification. The phased research agenda in Section 8.8 sets out how that might be done, and the work ought to come first, before the framework is leaned on to guide policy or practice.
